# Isolation and identification of bacteria causing mastitis in small ruminants and their susceptibility to antibiotics, honey, essential oils, and plant extracts

**DOI:** 10.14202/vetworld.2018.355-362

**Published:** 2018-03-26

**Authors:** Abeer Mostafa Abdalhamed, Gamil Sayed Gamil Zeedan, Hala Abdoula Ahmed Abou Zeina

**Affiliations:** Department of Parasitology and Animal Diseases, National Research Centre, 33 Bohouth Street, Dokki, 12622, Giza, Egypt

**Keywords:** black cumin, essential oil, honey, mastitis, onion, small ruminants, *Staphylococcus aureus*

## Abstract

**Aim:**

The present work aims to isolate and identify bacteria that cause mastitis in small ruminants and evaluates the antibacterial activity of some antibiotics, honey, essential oils, and plant extracts.

**Materials and Methods:**

A total of 289 milk samples were collected from udder secretions of sheep (n=189) and goat (n=100) from El-Fayoum, Beni-Suef, and Giza governorates. Screening subclinical mastitis (SCM) was done using California Mastitis Test (CMT); identification of the isolates was achieved using Gram’s staining, hemolytic pattern, colony morphology, and biochemical tests using Analytical Profile Index.

**Results:**

On clinical examination, the incidence of clinical mastitis (CM) was found to be 5.88% and 7% in sheep and goat, respectively. On CMT, SCM was found to be 25 (13.23%) and 11 (10%) in sheep and goat, respectively. Bacteriological examination of all milk samples found the presence of *Staphylococcus aureus* (SA) (31.1%), coagulase-negative staphylococci (CNS) (19.5%), *Escherichia coli* (EC) (8.3%), *Streptococcus* spp. (5.6%), *Klebsiella* spp. (3.77%), and *Pseudomonas* spp. (1.89%), while no bacteria were cultured from 81.66% of the samples. Identification of 9 isolates of CNS was achieved by using API staph test to *Staphylococcus epidermidis*, *Staphylococcus hominis*, *Staphylococcus cohnii*, and *Staphylococcus saprophyticus*. The highest bacterial resistance was found in EC (67.14%) followed by Kp (45.28%) and SA (26.57%).

**Conclusion:**

Onion and black cumin essential oils followed by Egyptian honey showed strong antibacterial effects against multidrug-resistant bacteria. Finally, our study proved that Egyptian honey, onion, and black cumin essential oils have a marked strong antibacterial effect against bacteria isolated from small ruminant mastitis, but still further extensive studies are needed to discover the therapeutic properties of these plant extracts and honey.

## Introduction

Mastitis is the inflammatory disease of mammary glands of small ruminants which may cause partial or full damage to udder, does not return to normal function, reduces body weight, and reduces the growth rate of their offspring [1]. In addition, there is economical loss due to treatment costs besides culling of sheep and goat [2]. Clinical and subclinical mastitis (CM and SCM) cause severe transitory inflammatory signs due to traumatic, pathological, and bacteriological changes in mammary glands lead to permanent blocking of milk ducts [3]. Predisposing factors such as poor management, bad hygiene, and teat injuries are known to be extrinsic factors to the entry of infectious agents [4].

Pathogenic microorganisms cause mastitis in sheep and goat which can only be detected in milk by laboratory tests. SCM is one of the most important diseases in small ­ruminants and considered a constant risk of infection for the whole herds and their environment. Hence, udder infection must be prevented or detected at an early stage not only to protect the farmer, but also to protect human consumer [5]. *Staphylococcus aureus* (SA)*, Staphylococcus* spp., and other environmental ­pathogens have been proven as the main causative agents of mastitis in dairy sheep and goats [6].

Antibiotics are used to treat mastitis in small ruminants. Furthermore, sensitivity tests can be done to ensure the adequacy of treatment [7,8]. The infectious multidrug-resistant bacteria have become a major health problem and limited the option for effective treatment in human and animal population worldwide [9].

Development of natural bacterial resistance to different types of antibiotics leads to arising of multidrug-resistant bacteria that causing financial and economic implication, due to non-effective treatment and spread drug-resistant bacteria from animals to animals. Although the new generation of advanced antibiotics was produced by pharmacological cooperation, still drug-resistant bacteria have been increasing. In addition, the adverse effects of antibiotics harm vital organs such as liver, kidney, pancreas, and spleen [10,11]. Honey has been used as a medication since ancient time, which is known as “traditional medicine” in various cultures, but nowadays, honey and herbal plant medicines have been used as therapeutic agents worldwide [12,13]. In the recent years, antibacterial activity of honey has been documented in several studies against Gram-positive bacteria (SA, *Bacillus subtilis, Bacillus cereus, Enterococcus faecalis*, and *Micrococcus luteus*) and Gram-negative bacteria (*Escherichia coli* [EC]*, Pseudomonas aeruginosa*, and *Salmonella typhi*), especially in Australian and New Zealand honey [13], European honey in general [14,15], Spanish honey [16,17], Brazilian honey [18], African honey [19], Egyptian honey [20,21], and Saudi Arabian honey [22,23].

Honey is produced from different sources and its efficacy against microbes depends on its types, sources, and concentration [24]. Honey has hydrogen peroxide and gluconic acid which originate from the dissolution of sugar by honey’s gluco-oxidase [24]. The action of honey is also linked with osmolality; in fact, its high sugar contents create a high osmotic pressure which is unfavorable to bacterial growth and proliferation. Honey has acidic pH ranging from 4.31 to 6.02, which plays a role in microbial inhibition. In addition, the components of honey have more than 181 constituents [25]. The antibacterial effect of New Zealand honey is very strong although it does not contain hydrogen peroxide or gluco-oxydase. A recent study reported that the compound of honey responsible for higher antibacterial activity is methylglyoxal which is effective against both Gram-positive and Gram-negative bacteria, with inhibitory effect ranging from 85.7% to 100% [26]. Essential oils and plant extracts are rich with a wide variety of metab­olite compounds (terpenoids, tannins, alkaloids, and flavonoids) [26]. Black cumin oil has been proven to be effective against a wide variety of microorganisms, but there are only a few studies available that describe the antimicrobial effects of essential oils [27]. Onion bulbs contain a good number of phytochemical properties, most of which are hydrocarbons and their derivatives. Several studies have proved that the plant extracts and essential oils have antimicrobial effects [28]. However, a large number of plant species have not been studied for their potential medicinal value [29].

The present study was aimed to isolate bacteria that cause CM and SCM in small ruminants and evaluate the antibacterial activities of some antibiotics, honey, essential oils, and plant extracts.

## Materials and Methods

### Ethical approval

All samples were collected as per standard ­sample collection procedure without giving any stress or harm to the animals. The present work was done according to the ARRIVE guidelines in accordance with the EU Directive 2010/63/EU for animal experiments and according to the guidelines of the National Institutes of Health Guide for the care and use of laboratory animals and the General Organization for Veterinary Services in Egypt.

### Animals and clinical data

Udder secretions were collected and clinical data were recorded from 289 sheep and goat with CM and apparently normal or SCM. The sheep and goat belonged to 20 flocks located in EL-Fayoum, Beni Suef, and Giza ­governorates were used for the study from September 2016 to July 2017. CM was present in one or both glands of sheep and goat. The California Mastitis Test (CMT) was used for detection of SCM in sheep and goat milk samples; it was carried out according to CLSI [30] and National Mastitis Council guidelines [31]. About 2 ml of milk samples from each quarter of dairy sheep and goat was collected in shallow cups of paddle, an equal amount of CMT reagent was poured into each cup; the sample was mixed by gentle circular rotation, and the results were interpreted based on dense gel formation. About 10 ml of milk sam­ples was collected in sterile plastic vials from mammary glands of sheep and goat according to Zeedan *et al*. [32] and National Mastitis Council [31].

### Plant essential oils and honey

Egyptian honey was purchased from the Ministry of Agriculture market, Cairo, Egypt. Essential oils of onion and black cumin were purchased from the unit of production and marketing of medicinal plants at the National Research Centre, Cairo, Egypt, and were extracted according to the guidelines of National Mastitis Council [31] and Taponen *et al*. [33]. The honey and essential oils were filtered with Seitz filter attached with a syringe. The filtrate from different samples was aseptically inoculated on nutrient agar and then incubated at 37°C for 24-48 h for sterility examination. The sterile samples extracted were stored in a sterile cupped brown bottle and kept in refrigerator at 4°C until use.

### Isolation of bacteria from milk samples

Bacterial strain isolation from milk samples was carried out following aseptic procedures as described by National Mastitis Council [31]. A loopful of milk sample was streaked on blood agar (Oxoid) supplemented with 5% sheep red blood cells and then subcultured on selective media, Mannitol Salt Agar, Salmonella Shigella Agar, Edwards medium, and MacConkey Agar. All plates were then incubated ­aerobically at 37°C for 24 h. The plates were examined for colony morphology, pigmentation, and hemolytic characteristics at 24-48 h. Catalase test was applied for distinguishing between staphylococci and other Gram-positive cocci, mannitol fermentation test, coagulase test (either positive or negative), hemolytic pattern, colony morphology and DNase activities, bacitracin (0.04 U), furazolidone (100 μg), novobiocin sensitivity, and biochemical tests. Analytical Profile Index-Staph (API-Staph Kit, bioMerieux, France) was used according to the manufacturer’s instructions to differentiate between *Staphylococcus* spp. according to methods described by Taponen *et al*. [33] and López-Malo *et al*. [34]. Furthermore, Gram-negative bacterial isolation was carried out according to the standard microbiological procedures described by CLSI [35].

The isolates were confirmed by biochemical tests and sub-cultured on differential and selective medium. The biochemical tests were oxidase activity, acid production (lactose sucrose and glucose fermentation), Indole production, Voges–Proskauer, hydrogen sulfide production and API-20 tests (API, bio Meraux, France).

### Determination of antimicrobial activities of plant extracts and honey

#### Disc diffusion method

This method was done according guidelines mentioned by guidelines mentioned by CLSI [30] and Perez *et al*. [36]. Bacterial isolates were diluted at a concentration of 10^6^ colony-forming unit (CFU), according to ­refer­ence to McFarland 0.5. The suspension was poured on plate with Mueller–Hinton agar after 1 h. 50 discs of 6 mm Whatman filter paper was obtained by punching and placing in bottle and sterilizing in hot air oven at 170°C for 30 min and served as a positive control of standard antibiotics. The filter paper with 6 mm diameter discs were impregnated with 20 µl of each essential oil and and diluted with honey (v/v); 1 g of honey was diluted in 1 ml of distilled water (v/v) and compared with reference antibiotics and solvent or double-distilled water as negative control aseptically placed with a sterile ­forceps on Mueller–Hinton agar plates. The plates were inculcated at 37°C for 24 h. Minimum bactericidal concentration was done according to the method described by CLSI [35] and Perez *et al*. [36]. The number of visible growth in minimum inhibitory concentration (MIC) assay was subcultured using a 10 µl inoculating loop onto a 5% sheep BAP and incubated at 37°C for 24 h.

#### Broth microdilution assays

The efficacy of essential oils, plant extracts, and Egyptian honey was determined by the microdilution method as described by CLSI [35,37]. Two-fold serial dilutions of essential oils (0.5, 0.25, 0.125, and 0.0625 mg/ml) in Muller Hinton broth were prepared in sterile polystyrene 96-well U-shaped plates. 100 µl of each essential oil and honey was used for estimation by MIC assays. The well contained medium alone or medium plus essentials oils without bacterial strains and medium plus bacterial strain as the control. Furthermore, antibiotics (penicillin G [PEN], erythromycin, ampicillin [AMP], gentamycin [GEN], vancomycin [VAN], cephradine [CED], clindamycin [CLI], tetracycline[TCN], and enrofloxacin [ENR]) were diluted with unique concentration of each antibiotic. Bacteria were diluted in the saline solution to about 1 × 10^6^ CFU/mL (0.5 Units of McFarland turbidity standard). After 24 h incubation, MIC was determined for total inhibition of growth compared with positive control and untreated medium plus essential oils and honey extracts which was carried out in triplicate.

### Statistical analysis

Data were subject to statistical analysis with means, standard division, and t-test at p<0.05 using SPSS for Windows 7 version 15.

## Results

Out of 289 samples of sheep and goat from ­different areas in three governorates, 18 CM cases were found and with unilateral or bilateral inflammatory response as well as systemic signs present in on mammary glands unilateral or bilateral as well as systemic signs present of sheep (11 [5.82%]) and goat (7 [7%]). The results of examination of samples from sheep and goat herds for SCM by CMT revealed that the percentage of positive CMT in sheep milk samples was 25 (13.23%) and goat milk samples 11 (10%). The overall percentage of SCM was 12.11% in sheep and goat milk samples as presented in Table-1. Bacteriological examinations of CM and SCM milk samples represented clinical and subclinical mammary infections of 15.55% from unilateral and bilateral mastitis.

**Table-1:** Examination of sheep and goat herds for CM and SCM by CMT at different locations.

Location	Number of examined sheep	Sheep, n (%)		Number of examined goat	Goat, n (%)	
	
		CM	SCM		CM	SCM
ElFayoum Governorate	79	4 (5.06)	13 (16.46)	35	2 (5.07)	3 (8.57)
Beni-Suef Governorate	50	2 (4.00)	4 (8.00)	45	3 (6.067)	2 (4.44)
Giza Governorate	60	5 (8.33)	8 (13.33)	20	2 (10.00)	5 (25.00)
Total number	189	11 (5.82)	25 (13.22)	100	7 (7.00)	10 (10.00)

SCM=Subclinical mastitis, CM=Clinical mastitis, CMT=California Mastitis Test

The highest number of bacteria isolated from milk samples were from SA, CNS, and EC. Gram-positive bacteria were differentiated by Gram stains and Catalase test. Furthermore, Gram-negative bacteria were confirmed by subculturing on differential media, selective media, and API-20 as shown in Table-2.

**Table-2:** Bacteriological examination of sheep and goat milk samples with clinical and subclinical mastitis.

Species	Total CM and SCM	Bacteriological examination, n (%)					

		Gram-positive bacteria			Gram-negative bacteria		
	
		SA	CNS	SE	EC	KS	PS
Sheep	17	4 (23.53)	2 (11.76)	1 (5.88)	1 (5.58)	1 (5.08)	0 (0)
	6	1 (16.67)	1 (16.67)	0 (0)	0 (0)	0 (0)	0 (0)
	13	5 (38.46)	3 (23.08	1 (7.69)	1 (7.69)	1 (20)	1 (20)
Goat	5	1 (20)	1 (20)	0 (0)	0 (0)	0 (0)	0 (0)
	5	1 (20)	0 (0)	1 (20)	0 (0)	0 (0)	0 (0)
	7	3 (43.86)	2 (28.57)	1 (14.29)	1 (14.29)	0 (0)	0 (0)
Total	53	16 (30.1)	9 (19.98)	4 (7.55)	3 (5.66)	2 (3.77)	1 (1.89)

SCM=Subclinical mastitis, CM=Clinical mastitis, CNS=Coagulasenegative staphylococci, SA=*Staphylococcus aureus*, SE=*Staphylococcus epidermidis*, EC: *Escherichia coli*, KS=*Klebsiella pneumoniae*, PS=*Pseudomonas* spp

Table-1 shows that the percentage of CM and SCM in sheep was 5.82% and 13.22%, respectively.

. The percentage of CM and SCM was 7% and 10% in goat, respectively. Total percentage of CM and SCM was 15.34% in sheep and goat.

Data represented in Table-2 clearly show that the percentage of bacteria isolated from CM and SCM after confirmation by biochemical characteristics and API20 was 30.1%, 19.9%, 7.55%, 5.66%, 3.77%, and 1.89% for SA, CNS, SE, EC, KS, and PS, ­respectively. A total of 35 bacterial isolates were recovered from 53 milk samples obtained from 289 milk samples of sheep and goat. Of the 29 isolates, 82.85% were Gram-positive and the remaining 17.15% were Gram-negative. The predominant bacterial isolates recovered were SA (30.1%), CNS (19.98%), SE (7.55%), and EC (5.55%) followed by *Klebsiella spp*. (3.77%) and *Pseudomonas* spp. (1.89%). Morphological characterization of isolated bacteria from CM and SCM of sheep and goat milk samples were Gram-positive, non-spore forming cocci, arranged in clusters, catalase positive, alpha hemolytic pattern on sheep blood agar, no change of color on mannitol salt agar, coagulase negative, bacitracin, furazolidone, novobiocin sensitive and API staph tests. CNS was identified to *Staphylococcus epidermidis*, *Staphylococcus hominis*, *Staphylococcus cohnii*, and *Staphylococcus saprophytic*.

Table-3 shows that the most effective antibiotics against Gram-positive bacteria isolated from small ruminant mastitis are; VAN effective against *S. aureus*, AMK, GEN and CRD effective against CNS. Also, Gram-negative bacteria were sensitive to GEN, AMK and CLI for *E. coli*, KS and PS respectively. Egyptian honey has some effect on microorganisms isolated from CM and SCM from sheep and goat at different concentrations. SA was the most affected microbe, while EC and *Klebsiella pneumoniae* (KP) were significantly the least affected microbes compared with the control sample. Essential oils (v/v) have significant antibacterial activity at lower concentrations compared with honey. In addition, different honey concentrations showed marked antibacterial effect compared with antibiotics on isolated bacteria. The efficacy of essential oils (onion and black cumin), antibiotics, and Egyptian honey against Gram-positive and Gram-negative bacteria with inhibitory zone diameter ranging from 13±0.56 to 28±0.23 and MIC value ranging from 3.25 to 25 mg/ml may be attributed to rich natural honey extract as well as plant extracts rich in contents of active compounds such as alkaloids and flavones. There was no significant variation in the antimicrobial activity of Egyptian honey and essential oil extracts of black cumin and onion at different concentrations.

**Table-3:** Antibacterial effect of antibiotics and essential oils, plant extracts, and honey against bacteria isolated from clinical and subclinical mastitis.

Antibacterial	IZD						MIC mg/ml					
	
	Gram+B			Gram−B			Gram+B			Gram−B		
			
	SA	CNS	SE	EC	KP	PS	SA	CNS	SE	EC	KP	PS
Extracts												
Onion oil	23±0.23	19±0.23	19±0.23	19±0.23	19±0.23	13±0.56	3.25	3.25	12.5	12.5	12.5	12.5
B. cum oil	26±0.25	22±0.25	24±0.25	26±0.25	23±0.25	15±0.75	3.25	3.25	6.25	6.25	6.25	6.25
E. Honey	28±0.23	14±0.25	14±0.25	16±0.25	16±0.25	17±0.56	3.25	3.25	6.25	6.25	6.25	6.25
Antibiotics												
PEN	6±0.26®	6±0.64®	5±0.36®	6±0.64®	7±0.25	6±0.25	12.5*	12.5	12.5	12.5	12.5	12.5
GEN	17±0.53	35±0.23	35±0.23	35±0.23	35±0.23	28±0.65	12.5	12.5	3.25	3.25	3.25	3.25
AMP	8±0.26	18±0.64	9±0.36®	8±0.64®	19±0.25	23±0.25	12.5*	12.5*	12.5	12.5	12.5	12.5
VAN	25±0.25	27±0.25	18±0.26	7±0.56®	6±0.25	6±0.25	12.5*	12.5	12.5	25	12.5	12.5
AMK	21±0.26	16±0.75	18±0.36	34±0.45	31±0.75	37±0.75	6.25*	6.25	6.25	12.5	12.5*	12.5
ERY	7±0.02	12±0.25	12±0.36	9±0.65	19±0.25	23±0.25	6.25	25	12.5	12.5	12.5	12.5
CRD	12±0.56	22±0.42	23±0.12	13±0.56	6±0.25	6±0.25	25	25	12.5	25	12.5	12.5
CLI	14±0.65	13±0.23	11±0.65	10±0.65	31±0.75	33±0.75	6.25	12.5	6.25	12.5	12.5	12.5
TCN	11±0.65	13±0.23	12±0.65	13±0.65	14±0.75	12±0.75	6.25	12.5	6.25	12.5	12.5	12.5
C. N												
DMSO	1±0.026	1±0.036	1±0.036	1±0.028	1±0.028	2±0.26	25	25	25	25	25	25
N. saline	1±0.20	1±0.16	1±0.16	1±0.19	1±0.19	1±0.26	25	25	25	25	25	25

CNS=Coagulasenegative staphylococci, SA=*Staphylococcus aureus*, SE=*Staphylococcus epidermidis*, EC: *Escherichia coli*, MIC=Minimum inhibitory concentration, IZD=Inhibition zone diameter, KP=*Klebsiella pneumoniae,* ®=Resistant, S=Suscetible, *ug/ml, onion essential oils, black cumin oils, Egyptian honey. PEN=Penicillin G, GEN=Gentamycin, AMP=Ampicillin, VAN=Vancomycin, AMK=Amikacin, ERY=Erythromycin, CRD=Cephradine, CLI=Clindamycin, TCN=Tetracycline, CN=control negative, DMSO=Dimethyl sulfoxide and N. saline=Normal saline

## Discussion

Mastitis in small ruminants is the most important disease and poses a serious socioeconomic impact worldwide [38]. The inflammatory response in the udder of sheep and goat is called mastitis [39]. CM can be detected by clinical examination such as inspection and palpation [40]. In the present study, the prevalence of CM in sheep and goat was 13.23% and 10% , respectively, and the combined prevalence was 12.11% in sheep and goats, which increased during the lambing season and subsequent weeks and also due to moving flocks to different pasture under various conditions and the variations in atmospheric and climate conditions, flock size, and management routines as shown in Table-1. This result is in agreement with that of Ergun *et al*. [41] who recorded 17% prevalence rate of SCM based on the results of CMT. The difference in prevalence rates may be attributed to a difference in the management system and study area. Reza *et al*. [42] and Wilson *et al*. [43] found that 90% of the differences in the goats’ somatic cell count (SCC) was not due to infection, but it was due to increasing the number of milking days which leading to reduced milk production. The higher percentage of positive CMT was 19-29% in Italy, 34.6% in Spain and 19% in the USA; this means that the positive result of CMT was not related to infection of goats. This is also supported by a high number of the negative bacterial count in milk samples. On the other hand, there is a disagreement with a study in Jordan which found that the prevalence of SCM between sheep was 66.9%, but in Brazil [44], the prevalence of SCM was 40.45% found in first partum Santa Ines ewes.

Table-2 shows that most of the bacteria isolated from sheep and goat milk samples were 30.1% of S. aureus. The highest percentage of *S. aureus* in sheep and goat are suggested to be reinfection of mammary glands and teat lesions which transferring during milking operation. SA is an important foodborne pathogen that may be transmitted to flocks during milking which is considered an important mechanism for the spread of this organism [45].

In flocks of sheep, the transmission of *S. aureus* infection among ewes could be results from herdsman during manual contact of udder, contaminated bedding material from infected ewes and lambs sucking ewes other than their dam [46].

Many studies in different regions worldwide have investigated the prevalence of CNS-causing mastitis [47-49]. The occurrence of mammary infections with CNS has been documented [48]. In a similar study that was conducted in Norway, the prevalence of CNS was 16% [49], while in Germany, CNS was isolated from 9% of milk samples of 80 dairy herds [49]. The identified species of CNS according to the 9 isolates from CM and SCM from sheep and goat were SE*, S. hominis, S. cohnii*, and *S. saprophytic* which may be due to environmental hazard [47].

CNS isolated from mastitis milk samples were *S. hyicus, S. simulans, SE, S. hominis, S. cohni, S. saprophyticus, Staphylococcus sciuri, Staphylococcus capitis*, and *Staphylococcus lentus* [50,51].

The most frequent pathogen isolated from CM and SCM in our study were *Streptococcus* spp. 7.5%, *E. coli*, 5.66%, *K. pneumoniae*, 3.77%, and *Pseudomonas* spp. 1.89%, which is similar to the proportions found in other research studies on sheep and goat [52,53]. The numbers of clinical cases caused by Gram-negative bacteria causing systemic signs. The treatment of mastitis with antibiotics usually initiated by farmer or herdsman and not by veterinarian in most of the inflammatory diseases of mammary gland which leading to progression of SCM to CM. SCM might result from inappropriate treatment of CM and as a result, infections remain undetected for a long time. Table-3 shows that the antibiotics PEN and AMP, were less effective than AMK against *E. coli*, *K. pneumoniae* and *S. aruers* respectively. The most common Gram-negative EC and KP strains have multidrug resistance to most common antibiotics used. While amikacin has been recorded as the most effective antibiotic against all isolated bacteria with inhibition zone diameter of 37±0.75 and 30±0.56 mm, PEN, AMP, and TCN were found to be less effective against all isolated bacteria from mastitis milk of sheep and goat. This is due to increased indiscriminate and frequent use of those antibiotics which leads to the development of antibiotic-resistant bacteria which in turn necessiates development and search for new sources as antimicrobial agents, and this finding is in agreement with Aumeeruddy-Elalfi *et al*. [54] and Azim [55]. They found that the Gram-negative and *Pseudomonas* spp. are more resistant to antibiotics than Gram-positive bacteria due to lipopolysaccharide layer of Gram-negative bacteria in outer membrane which acts as a highly hydrophobic, protected, and induced barrier against hydrophobic molecules, this explanation is in consistent with Eja *et al*. [56]. The current issue associated with multidrug-resistant bacteria is a worldwide problem, requiring research on natural and herbal plants to solve this problem. Herbal green medicine has been practiced worldwide for centuries, particularly for therapeutic purposes [57]. Essential oil extract, onion, and black cumin have stronger antibacterial activity which may be due to their volatile oil materials that cause membrane dysfunction of bacterial cell wall leading to mitochondrial cell damage; the difference between their effects may be due to the quantity of the phenolic compound, eugenol. Egyptian honey has inhibitory effect against isolated bacteria which may be due to its complex composition including hydrogen peroxide and bee-derived enzyme glucose oxidase which has a bactericidal effect, this result is in agreement with Nelson *et al*. [28] and Saad *et al*. [58]. Egyptian honey is composed of oligosaccharides, glycopeptides, phenol, fatty acids, lipids, amylases, ascorbic acid, peroxidases, and fructose, all these elements potentiate each other as antimicrobial agents [59]. Essential oil and plant extracts have strong antimicrobial properties at different concentrations of 50%, 20%, and 10%, and this finding is in consistent with Hegazi [59] and Lemos *et al*. [60]. Finally, our results proved that the essential onion oils, black cumin, and honey are safe and efficient, and have strong antibacterial effect against Gram-positive and Gram-negative bacteria.

## Conclusions

It can be concluded that the CM and SCM in dairy sheep and goats are caused by SA followed by CNS with other bacteria such as EC*, Streptococcus* spp., *Klebsiella* spp., and *Pseudomonas* spp., which cause infection in udder of sheep and goat. CNS is emerging as a minor mastitis pathogen in Egyptian dairy sheep and goat. Such species can cause decreased milk production. This pathogen reflects the environmental hazard. Chloramphenicol and ciprofloxacin were identified as the most effective antibiotics for the treatment of intramammary infections. From this perspective, natural products such as essential onion oils, black cumin, and honey have found to be efficient and have a strong antibacterial effect against Gram-positive and Gram-negative bacteria. However, they depend on quality, type, and source. Essential oils of onion and black cumin have shown a broad-spectrum antimicrobial activity. Further, molecular epidemiological studies for exploring the different types of CNS-causing mastitis in small ruminants should be done. Furthermore, we still need deep, extensive studies on honey and essential oil extracts for exploration of the active ingredient responsible for the antimicrobial effect.

## Authors’ Contributions

GSGZ and AMA involved in conception of the research idea, planned the study design, performed data collection and involved in sample collection, laboratory work, interpreted the data results, and helped in manuscript preparation. HAAA provided some laboratory materials, and she helped in the drafting of the manuscript. All the authors read and approved the final manuscript.
